# Unmet Dental Care Needs among Korean National Health Insurance Beneficiaries Based on Income Inequalities: Results from Five Waves of a Population-Based Panel Study

**DOI:** 10.3390/healthcare8020124

**Published:** 2020-05-05

**Authors:** Minsung Sohn, Xianhua Che, Hee-Jung Park

**Affiliations:** 1 Department of Health and Care Administration, The Cyber University of Korea, Seoul 03051, Korea; minsinge@cuk.edu; 2 Department of Health Policy Research, Daejeon Public Health Policy Institute, Daejeon 282, Korea; chexianhua719@gmail.com; 3 Department of Dental Hygiene, College of Health Science, Kangwon National University, Gangwon-do 25945, Korea

**Keywords:** dental care access, health insurance, unmet dental care needs, South Korea

## Abstract

This study investigates whether self-employed beneficiaries experience greater difficulties in accessing dental care than insured employees based on their income level. This analysis uses 2011–2015 data from the Korea Health Panel, a population-based and nationally representative sample, covering 7083 participants aged 18 years and older. We measured barriers to dental access based on unmet needs or the inability to receive necessary dental care owing to the past year’s economic burdens. The type of health insurance and household income are considered independent variables. We applied multiple panel logistic regressions and two-panel logistic regression models with a fixed-effects approach to analyze the data. Self-employed beneficiaries were 1.16 times (95% confidence interval (CI) = 1.08–1.24) more likely to experience unmet dental needs than were insured employees. Insured employees and self-employed beneficiaries belonging to the lowest income bracket were 1.76 times (95% CI = 1.53–2.03) and 2.33 times (95% CI = 1.89–2.87) more likely to have unmet needs than those in the highest income bracket. Self-employed beneficiaries were 1.31 times (95% CI = 1.21–1.43) more likely to experience unmet dental needs caused by economic burdens than are insured employees. Insured employees of the lowest income quintile were 4.15 times (95% CI = 3.41–5.05) more likely to experience unmet needs caused by economic burdens, while the odds ratio for self-employed beneficiaries was 5.47 (95% CI = 4.05–7.39). Our findings indicate gaps in unmet dental needs between self-employed beneficiaries and insured employees. The government should adopt strategies to reduce unmet needs among marginalized groups and redefine the role of national health insurance.

## 1. Introduction

National Health Insurance (NHI) was introduced in South Korea in 1977, and had universal coverage in 1989, with the objective of providing comprehensive medical services to all citizens. This was done by lowering economic barriers and improving access to medical care. NHI provides benefits for prevention, diagnosis, disease and injury treatment, rehabilitation, and dental care [1,2]. NHI, an independently operated national health insurance system, is a social insurance and medical aid program offering public medical assistance to the absolute poor, whose income is below the minimum cost of living [2]. As of January 2018, 50.94 million people (97.1%) out of South Korea’s total population of 52.44 million were covered by health insurance, with 1.5 million (2.9%) beneficiaries [3].

Health insurance beneficiaries are divided into two groups—insured employees (i.e., regular workers) and insured self-employed (i.e., unemployed or self-employed)—based on economic activity or income level. Insured employees are regular wage workers, while the insured self-employed group mostly consists of unemployed workers without income or self-employed workers without employees. Under South Korea’s universal healthcare system, the cost-sharing rate for health insurance beneficiaries to utilize inpatient and outpatient services, prescription drugs, and dental care is equal (i.e., co-payment for health insurance beneficiaries is 30%–50%) [4]. Irrespective of the equal coverage and cost-sharing structures, the varying NHI premiums under an income-proportional fixed-rate system for employees and the self-employed cause an equity burden—employers incur half the premium for insured employees, whereas the self-employed must pay the full premium. This results in the unequal distribution of medical care, intensified by economic hardships among the latter [4,5].

Consequently, the self-employed—who face a disproportionate premium burden—are frequently unable to access healthcare services and are likely to report greater unmet healthcare needs than insured employees are. The inequality in health status between insured employees and the self-employed is particularly observable in healthcare access [6,7]. Further, the income level of self-employed beneficiaries is likely to aggravate the unequal access to and use of healthcare services [8].

In the case of dentistry, dental care currently accounts for approximately 20% of the dental service expenditures of public programs in South Korea [9]. The proportion of out-of-pocket payments in Korea is 84%, while that for the Organization for Economic Co-operation and Development (OECD) averages 55%; in fact, Korea’s proportion is about 3.5 times that of Japan (24%) and double that of the United States (42%) [10]. Such barriers in access to dental services increase the rate of unmet dental care needs, particularly for those with low socioeconomic status [9,11,12], as defined by income level, education, occupation, and/or area of residence. According to the Korean National Health and Nutrition Examination Survey (2016), the annual rate of untreated dental conditions among the adult population (19 years and above) is approximately 26% [13], which is significantly higher than that reported for European countries, such as Switzerland (4.6%), the United Kingdom (2.3%), Portugal (14.2%), and Spain (7.4%) [14]. Previous studies have confirmed that vulnerable and deprived populations in many countries experience considerable barriers to dental care [12,14,15,16]. Among those affected, older adults [17,18], individuals with lower income [11,15], and education levels [9,19], those without dental insurance [20,21], and the self-employed [22] are less likely to visit a dentist. Lee et al. reported social inequalities in dental care access for periodontal treatments between insured employees and self-employed beneficiaries, with the latter less likely to receive such treatment [23]. Park and Che reported cases of self-employed beneficiaries who had been unable to obtain the necessary dental care for three consecutive years [22]. Moreover, Choi et al. recently suggested that self-employed workers are more likely to face barriers in access to dental care as a result of economic hardships than full-time permanent workers are [24]. Despite such empirical evidence, little attention has been paid to income-related inequalities in unmet needs based on the types of health insurance beneficiaries. We hypothesize that self-employed beneficiaries experience greater difficulties in accessing dental care than insured employees based on their income level. Thus, we explore the role of income-related inequalities in unmet care needs on the basis of household income and the type of health insurance beneficiary.

## 2. Materials and Methods 

### 2.1. Study Design

Our data were sourced from the Korea Health Panel (KHP) survey administered by the Korea NHI Service and the Korea Institute for Health and Social Affairs. We used data from the sixth to tenth wave conducted during 2011–2015 to build a longitudinal dataset for this study. The KHP surveys offer information on medical utilization, medical expenditures, health status, and health behaviors. They are nationally representative of households in South Korea and are conducted through computer-assisted personal interviewing. Before analyzing the panel survey, we accounted for varying sample sizes from 2011 to 2015 and missing values of variables to ensure a balanced dataset.

### 2.2. Study Population

**Figure 1:**
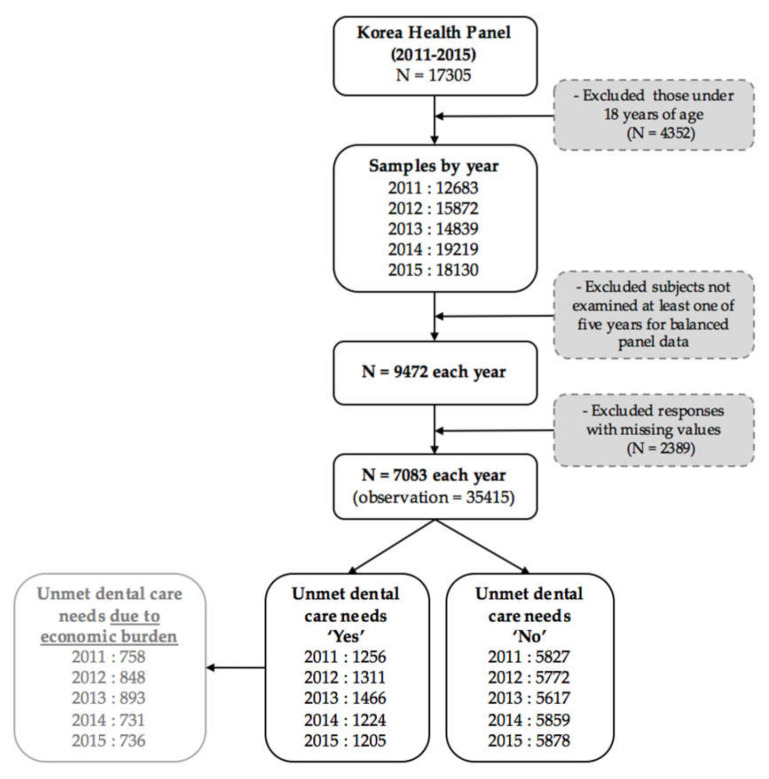
The flow of sample selection in this study.

The sampling was performed using a two-stage, stratified, cluster extraction method with probability proportionality. We excluded 4352 people under 18 years of age from among the 17,035 people who responded to the survey during our five-year sample period. Then, we selected 9472 subjects each year, by excluding subjects who were not examined in all five years, to ensure a balanced panel data set. Excluding responses with missing values gave us a final sample of 7083 adults each year and 35,415 observations. The annual average frequency of people experiencing unmet dental care needs over those five years was 18.24% of the sample (*n* = 1292), and 12.04% (*n* = 853) experienced unmet dental care needs owing to economic burdens. (See Figure 1)

### 2.3. Variables

#### 2.3.1. Dependent Variables

We employed two variables to measure barriers to dental care access. In the KHP survey, respondents were asked the question, ‘Have you ever experienced unmet dental needs within the past year?’ Participants who checked ‘yes’ were asked to specify the reason(s) for unmet dental needs from a list. Those who stated ‘economic burdens’ were considered to have unmet needs owing to economic hardships in the previous year. We categorized the outcome as ‘yes’ if the subjects reported unmet dental needs or an inability to receive necessary dental care owing to economic burdens and ‘no’ otherwise.

#### 2.3.2. Independent Variables

We considered the type of health insurance and household income as key independent variables. The type of NHI beneficiary was grouped into (1) health insurance for employees (employee beneficiaries) and (2) health insurance for the self-employed (self-employed beneficiaries). The KHP data provide a nominal household income quartile variable. This variable is calculated based on equivalent income according to equalized gross annual household income (equivalent household income = monthly household income/square root of the number of household members); the first quintile is the lowest 20%, and the fifth quintile is the highest 20% which was taken as a reference.

#### 2.3.3. Covariates

We organized the covariates into three groups on the basis of Anderson’s health behavioral model with predisposing, enabling, and need factors [25]. Predisposing factors included gender, age group, and education. Gender was dummy coded (Male = 1; Female = 2). Age was classified into three groups: 18–44 years, 45–64 years, and 65 years or above. Education level was also divided into three groups: respondents with a college or higher-level degree were treated as the reference group and coded as 1, high-school graduates were coded as 2, and middle-school graduates or those with a lower-level degree were coded as 3. Employment status was considered to be an enabling factor and grouped into (1) permanent employment, (2) temporary employment, (3) self-employment, and (4) economically inactive (e.g., housewives, students, or early retirees). We selected self-rated health, chronic diseases, and disability, known to affect unmet dental care need as need factors [22,26]. Self-rated health was divided into three groups: respondents who reported their health to be excellent, fair, or poor were coded as 1, 2, and 3, respectively. Further, respondents with chronic diseases (e.g., hypertension, diabetes, or arthritis) were coded as 1, and those without such diseases were coded as 2. Finally, respondents were coded as 1 if they had either a physical disability (e.g., auditory disorder, visual impairment, or brain disorder) or a mental disability (e.g., intellectual disability, mental disorder, or autistic disorder); if not, they were coded as 2.

### 2.4. Statistical Analysis

We conducted a trend analysis and panel logistic regression analyses, using weights to make the data nationally representative. All analyses were conducted in two groups by types of NHI beneficiaries (employees and self-employed) through stratification analysis. First, we conducted a trend analysis of unmet dental care needs by income quintile group and health insurance beneficiary from 2011 to 2015. Second, we performed a chi-squared test to describe the general characteristics of the study population and to check for differences between the types of NHI beneficiaries based on unmet dental care needs and the inability to receive necessary dental care owing to economic burdens. Third, we ran multiple panel logistic regressions to assess the independent association between unmet dental care needs and income level. We then applied two-panel logistic regression models with a fixed-effects approach. The fixed-effects logit model estimation focuses on within-individual comparisons across the five waves by controlling for unmeasured time-variant confounders. In model 1, we used health insurance type and income, as well as the effects on unmet dental care needs and the inability to receive necessary dental care owing to economic burdens. In model 2, we included covariates (i.e., gender, age, education level, employment status, health status, whether an individual had a chronic disease, and whether an individual had a disability) to explore the effect of income level on the odds ratio of the outcomes between employee and self-employed beneficiaries. 

All *p*-values were two-tailed with a significance level of < 0.05. All analyses were conducted using STATA 15 (StataCorp LP., Texas, USA).

### 2.5. Ethics Approval Consent to Participate

This survey did not require formal ethical approval under national laws. We used only public data from the KHP, which did not include any personally identifiable data. Ethical and governance approvals were granted by the Korea Institute for Health and Social Affairs. All participants gave written informed consent to take part before they were allowed to complete the survey.

## 3. Results

The general characteristics of the study population are presented in Appendix A, Table A1. Our study comprises 71.6% insured employee beneficiaries and 28.4% self-employed beneficiaries in latest year (2015). A total of 58.6% of employee beneficiaries and 57.1% of self-employed beneficiaries were female. As for the education level, the number of individuals with a college or higher degree was greater among employee beneficiaries (43.8%) than in the self-employed group (30.4%). The proportions of insured employee and self-employed beneficiaries categorized under the highest income quintile were 30.6% and 20.7%, respectively. As for employment status, the economically inactive accounted for the highest proportion of the self-employed group. Further, 88.7% of insured employee beneficiaries and 88.1% of self-employed beneficiaries reported fair or excellent health status, while the numbers of those with a chronic disease was marginally higher among the self-employed beneficiaries (11.5%). Finally, 4.5% of insured employee beneficiaries and 4.0% of self-employed beneficiaries reported having a disability.

**Figure 2:**
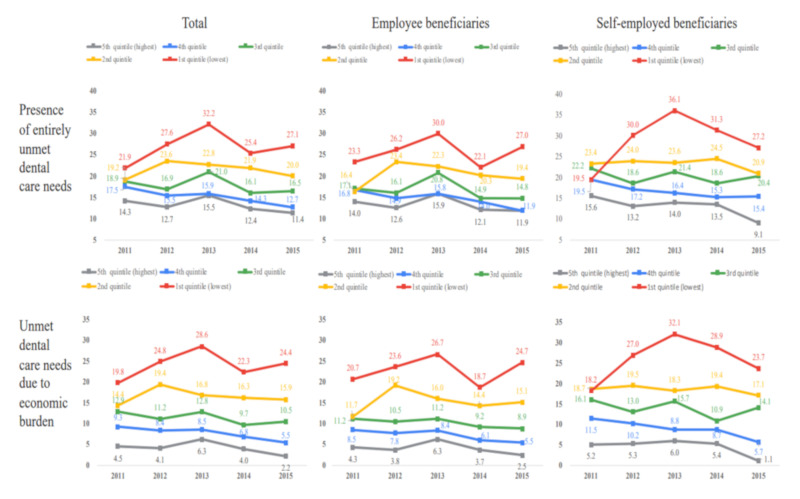
Entirely unmet dental care needs by income quintile group and the type of NHI beneficiary.

Figure 2 shows the proportion of unmet dental care needs and the inability to receive the necessary dental care owing to economic burdens by income quintile group from 2011 to 2015. The rate of unmet dental care for the highest income group (fifth quintile) increased from 14.3% in 2011 to 15.5% in 2013 and declined thereafter (11.4% in 2015). The lowest income quintile reported the highest rate of unmet dental care needs, the greatest inability to receive necessary dental care owing to economic burdens, and the greatest rate of change.

In the insured employee group, the rates of unmet dental care needs and the inability to receive necessary dental care owing to economic burdens both fell for the highest income quintile (14.0% to 11.9% and 4.3% to 2.5%, respectively) as well as for the fourth income quintile (16.8% to 11.9% and 8.5% to 5.5%, respectively). In the lowest income group, the rate of unmet dental care increased from 23.3% in 2011 to 30.0% in 2013, declined to 22.1% in 2014, and rose to 27.0% in 2015. The rate of unmet dental care needs owing to economic burdens also increased from 20.7% to 26.7% in 2013, declined to 18.7% in 2014, and rose to 24.7% in 2015.

The second to fifth income quintiles for the self-employed reported similar proportions of unmet dental care needs and unmet needs owing to economic burdens to those of insured employees. However, in 2013, the lowest income group of self-employed beneficiaries reported higher rates of unmet dental care needs (36.1%) and inability to receive necessary dental care owing to economic burdens (32.1%) than insured employee beneficiaries did (30.0% and 26.7%, respectively). The rate of unmet dental care needs in the lowest income group increased from 19.5% in 2011 to 36.1% in 2013 and declined to 27.2% in 2015. Unmet dental care needs owing to economic burdens increased from 18.2% to 32.1% and declined to 23.7% in 2015.

Table 1 presents the results of the chi-squared tests. The results show that 14.7% of insured employee beneficiaries and 17.7% of self-employed beneficiaries reported unmet dental care needs in the previous 12 months. Those aged 65 years or above and those with lower educational status, low-income levels, poor health conditions, or chronic diseases reported high rates of unmet dental care needs in both the insured employee and self-employed groups.

Table 2 presents the results of the chi-squared tests of unmet dental care needs owing to economic burdens. The results also show that 8.2% of insured employees and 11.2% of the self-employed had experienced unmet dental care needs owing to economic burdens. In both groups, those especially vulnerable to not receiving the necessary dental care owing to economic burdens were female participants aged 65 years or older and with a lower educational status, low income, or poor health status.

Table 3 presents the results from a logistic panel analysis with the fixed-effects approach. The self-employed beneficiaries were 1.17 times (95% CI = 1.10–1.25) more likely to have unmet dental care needs than insured employees were in model 1. As stated above, in model 2, we added covariates—gender, age, education level, employment status, health status, whether an individual has a chronic disease, and whether an individual has a disability—and found that self-employed beneficiaries were 1.16 times (95% CI = 1.08–1.24) more likely to have unmet dental care needs than employee beneficiaries were. As for income level, those in the lowest income bracket (1st quintile) were 2.36 times (95% CI = 2.14–2.61) more likely to have unmet needs than those in the highest income bracket (5th quintile) were. When we stratify participants by type of health insurance beneficiary in model 2, respondents in the lowest income bracket were 1.76 times (95% CI = 1.53–2.03) and 2.33 times (95% CI = 1.89–2.87) more likely to have unmet needs than those in the highest income quintiles were for insured employee and self-employed beneficiaries, respectively.

Table 4 lists the results for the inability to receive necessary dental care owing to economic burdens based on the type of beneficiary. After adjusting for all covariates, we found that the self-employed were 1.31 times (95% CI = 1.21–1.43) more likely than employees to experience an inability to receive necessary dental care owing to economic burdens. The lowest income group experienced an inability to receive necessary dental care owing to economic burdens more often than the highest income group did (OR = 4.53; 95% CI = 3.85–5.33). Further, those belonging to the lowest income and insured employee groups were 4.15 times (95% CI = 3.41–5.05) more likely to experience an inability to receive necessary dental care owing to economic burdens than the highest group was, and the odds ratio for self-employed beneficiaries was 5.47 (95% CI = 4.05–7.39).

## 4. Discussion

This study examined income inequalities in unmet dental care needs between insured employee beneficiaries and self-employed beneficiaries of NHI, using representative panel sample data for 2011–2015. After adjusting for potential confounders, we find that self-employed beneficiaries were at a significantly higher risk of limited access to dentists (OR = 1.16, 95% CI 1.08–1.24) than insured employees are. In addition, self-employed beneficiaries were more likely to experience an inability to receive necessary dental care owing to economic burdens than insured employees were (OR = 1.31, 95% CI = 1.21–1.43). These findings were consistent with those in the recent Korean literature, which suggests that over the past three years, self-employed beneficiaries experienced unmet dental care needs 1.69 times more than insured employees did [22]. Further, the present study provided evidence on the relationship between disparities and dental service access in South Korea’s NHI system. Our findings may also be congruent with the existing evidence on lower access to dental visits for periodontal treatments among self-employed beneficiaries [23].

A critical question this study attempts to answer is why self-employed beneficiaries in South Korea experience greater unmet dental needs than insured employees do, despite the similarities in individual plans in terms of service coverage and the extent of deductibles and co-payments. There are several possible explanations for such discrepancies. One is that insured employees and self-employed beneficiaries inherently differ in socioeconomic status, with significant variations in social class and premium levels. Self-employed beneficiaries who are older, less educated, and have lower incomes tend to avoid visiting a doctor despite their perceived need for care [5,6]. In particular, the elderly who fall in the self-employed beneficiary group, and report low incomes owing to retirement, have poorer health conditions resulting from their tendency to forgo healthcare [8]. Thus, it is likely that the elderly population among self-employed beneficiaries may have been negatively affected by avoiding utilizing dental services [22]. Further, prior evidence suggests that premium rates can impact medical utilization and/or attitudes toward healthcare [6,8]. In South Korea, self-employed beneficiaries report higher premiums than insured employees do because of differential income-based premiums, which, in turn, become a barrier to accessing healthcare. Thus, it is probable that certain self-employed beneficiaries choose to forgo healthcare or dental care owing to their cost, further hindering their ability to seek care.

Another potential explanation is that self-employed beneficiaries tend to work in dangerous environments with lower wages and more limited social benefits than regular wage workers do [8,27]. In South Korea, self-employed individuals account for more than 30% of the labor market [28], of which 80% are self-employed insurance beneficiaries [8]. Previous studies demonstrate that precarious employment conditions with lower purchasing power and job insecurity result in poor health status or outcomes; moreover, such workers generally experience significant barriers to medical care access [29,30,31]. Recently, Choi et al. clearly verified that self-employed status, defined as workers who manage their own businesses or carry out professional matters under their own responsibility, affects unmet dental care needs [24]. This result suggests that self-employed workers may be associated with lower income classes and are more exposed to the danger of working in poverty. Another study also highlights the greater likelihood of precarious workers—including self-employed persons with low income—having limited access to dental care and greater unmet dental care needs compared to their non-precarious counterparts [32]. Thus, a possible explanation can be proposed related to the working conditions of self-employed beneficiaries with lower incomes and their relationship with dental care access.

A second noteworthy aspect of this study is that, by comparing the five quintiles (Table 4) and adjusting for all variables, the analysis showed whether unmet dental needs were greater among insured employee beneficiaries (OR = 1.76; 95% CI = 1.52–2.14) or self-employed beneficiaries (OR = 2.33; 95% CI = 1.89–2.87) in the lowest income groups. The odds of having unmet needs due to economic burdens were 4.15 (95% CI = 3.41–5.05) for insured employees and 5.47 (95% CI = 4.05–7.39) for self-employed beneficiaries when comparing the lowest and highest income groups (Table 4). Our findings suggest that insured beneficiaries experienced inequality in dental care access based on their low incomes. In other words, insured beneficiaries at the bottom of the socioeconomic pyramid had significantly lower dental care security in terms of medical expense guarantees, and, thus, the NHI system’s objective of medical security for all participants is not being met. These findings move beyond previous research showing that income level is inversely associated with forgoing dental care for disadvantaged populations by documenting the importance of income inequalities in dental care service across the two beneficiary types in South Korea [11,12,15]. In concordance with the findings of a previous study of NHI and Medical Aid beneficiaries in South Korea [33], our study highlights the vulnerability of insurance beneficiaries.

For example, South Korea’s national health policy has turned its attention toward publicly funding dental care programs under the NHI system. Major dental insurance policies currently cover the cost of preventive periodontal scaling for individuals aged 20 years or above (as of July 2013). In addition, NHI began covering the cost of sealant in 2009, resulting in higher cost-sharing (70%–90%) and lower out-of-pocket expenses (30%–10%), as well as dental implants and dentures for the elderly (reduced deductibles and co-payment) in 2017. Despite this sizable commitment, dental care services are neglected by public insurance schemes. A significant proportion of beneficiaries do not take advantage of this coverage; moreover, the coverage rate of health insurance is about 25%–35% [9], which is generally limited to preventive care and restorative treatment. Previous observations suggest that since the socially disadvantaged are more likely to need dental care services that have high deductibles, such as prostheses or implants, patients with lower incomes tend to delay dental treatments until their oral health conditions become critical [11,34]. In addition, certain low-income individuals receive dental care because their conditions have significantly deteriorated as a result of insufficient early intervention and decisions to use medical services that are minimally covered by their insurance scheme and have low deductibles for outpatient visits [9,35]. Thus, we assured that unmet dental needs are most marked among the lowest-income insured households.

Our results highlighted that income-related inequalities in dental care access persist even in the presence of insurance coverage, and this can be attributed—at least in part—to gaps in universal insurance coverage. In other words, a considerable proportion of the insured low-income population is at risk of forgoing dental care under South Korea’s dental care system. Thus, it is necessary for health insurance schemes to ensure equality in access to dental care and to tackle the issue of high out-of-pocket costs incurred by the socially disadvantaged [33]. Increasing the scope of insurance coverage for certain dental services may eliminate economic barriers and improve access for low-income families and/or the working poor.

However, this study is not free from limitations. We focused on self-reported unmet dental care needs, and, thus, the responses were subject to recall bias. Unmet healthcare needs have often been defined as the inability to obtain the necessary healthcare, measured by barriers to patient care or the lack of or delayed healthcare [28]. However, these may be based on subjective perceptions, influenced by respondents’ socioeconomic characteristics and their objective dental health status. We reference previous studies to determine factors influencing unmet needs—in particular, cost barriers associated with the higher prevalence of dental treatment needs (e.g., untreated decay, missing teeth, or poor oral health [11,34]; in doing so, we consider only subjective general health conditions limited to the available data. The reasons for unmet dental needs are multifactorial and complex, including the lack of time, the unavailability of dentists, and distance to care facilities [15,23]. These reasons have not been sufficiently discussed in the present study. Moreover, a previous study indicated that the determinants of dental care deprivation effect have a distinctly different intensity in men and women [16]. The possibility of not receiving necessary dental treatment affects women significantly more than men [11,16]. Therefore, it is necessary to assess the effect of gender difference on inequality in unmet dental care needs related to the accessibility of dental care.

Despite these limitations, a key strength of this study is its use of a large sample size and data from multiple years to gain a better understanding of unmet dental needs, based on income levels among employees and self-employed beneficiaries under the health insurance program. These findings have important policy implications for South Korea. The results highlight the need to reduce income discrepancies in unmet dental care needs among employees and self-employed beneficiaries under NHI. Our findings could help in the formulation of policies to improve the use of and access to public health services for the insured population near the poverty line under the universal healthcare system.

## 5. Conclusions

Our study elucidates gaps in access to dental care services by income level and health insurance status in South Korea. The findings suggest the need to implement policies to improve efforts aimed at reducing unmet dental care needs among the socioeconomically disadvantaged beneficiaries of the NHI system, with a focus on the self-employed. In addition, increasing dental care benefits under NHI is an essential step to achieve universal coverage of South Korea’s population.

## Figures and Tables

**Table 1:** The difference rate of unmet dental needs between the type of NHI beneficiary, 2015.

Variables	Total			Employee Beneficiaries			Self-Employed Beneficiaries		
	N	Weighted %	*p*-Value	N	Weighted %	*p*-Value	N	Weighted %	*p*-Value
Total	1205	15.5%	-	824	14.7%	-	381	17.7%	-
Gender									
Male	469	15.5%	0.186	311	13.7%	0.181	158	17.2%	0.668
Female	736	16.0%		513	16.9%		223	18.0%	
Age									
18–44	218	11.1%	<0.001	149	10.3%	<0.001	69	13.6%	0.001
45–64	453	15.8%		291	14.9%		162	17.8%	
More than 65	534	24.4%		384	24.3%		150	24.5%	
Level of education									
≥College	260	11.7%	<0.001	203	11.7%	<0.001	57	11.3%	<0.001
High school	349	14.5%		217	13.2%		132	17.3%	
≤Middle school	596	23.5%		404	22.8%		192	25.0%	
Income level									
5th quintile (highest)	185	11.4%	<0.001	147	11.9%	<0.001	38	9.1%	<0.001
4th quintile	216	12.8%		150	11.9%		66	15.4%	
3rd quintile	248	16.5%		158	14.8%		90	20.4%	
2nd quintile	281	20.0%		176	19.4%		105	20.9%	
1st quintile (lowest)	275	27.1%		193	27.0%		82	27.2%	
Employment status									
Permanent employment	155	11.1%	<0.001	145	10.8%	<0.001	10	22.8%	0.806
Temporary employment	238	16.4%		171	15.8%		67	18.2%	
Self-employed	223	18.4%		90	18.5%		133	18.3%	
Unpaid family worker	68	16.8%		35	16.5%		33	17.2%	
Economically inactive population	521	16.8%		383	16.9%		138	16.4%	
Health status									
Excellent	300	10.5%	<0.001	211	10.2%	< 0.001	89	11.3%	<0.001
Fair	609	17.8%		401	16.5%		208	21.4%	
Poor	296	26.1%		212	25.8%		84	26.8%	
Chronic diseases									
No	256	4.6%	<0.001	170	4.4%	<0.001	86	5.2%	0.009
Yes	949	10.9%		654	10.3%		295	12.5%	
Disability									
No	1102	15.2%	<0.001	746	14.3%	0.001	356	17.4%	0.157
Yes	103	23.5%		78	23.4%		25	23.6%	

Note: We present weighted percentages of who answered ‘yes’ were asked for unmet dental needs by each category. Source: Korea Health Panel.

**Table 2:** The different rates of unmet dental needs and those due to economic burdens between the type of NHI beneficiary, 2015.

Variables	Total			Employee Beneficiaries			Self-Employed Beneficiaries		
	N	Weighted %	*p*-Value	N	Weighted %	*p*-Value	N	Weighted %	*p*-Value
Total	736	9.0%	-	496	8.2%	-	240	11.2%	-
Gender									
Male	263	7.7%	0.004	167	6.7%	0.002	96	10.6%	0.485
Female	473	9.9%		329	9.3%		144	11.7%	
Age									
18–44	68	3.5%	<0.001	39	2.8%	<0.001	29	6.2%	0.001
45–64	254	9.3%		158	8.4%		96	11.3%	
More than 65	414	19.9%		299	19.9%		115	19.7%	
Level of education									
≥College	97	4.2%	<0.001	72	4.0%	<0.001	25	5.2%	<0.001
High school	177	7.6%		111	6.7%		66	9.5%	
≤Middle school	462	18.8%		313	18.2%		149	20.1%	
Income level									
5th quintile (highest)	38	2.2%	<0.001	32	2.5%	<0.001	6	1.1%	<0.001
4th quintile	94	5.5%		69	5.5%		25	5.7%	
3rd quintile	154	10.5%		95	8.9%		59	14.1%	
2nd quintile	209	15.9%		128	15.1%		81	17.1%	
1st quintile (lowest)	241	24.4%		172	24.7%		69	23.7%	
Employment status									
Permanent employment	54	3.7%	<0.001	49	3.5%	<0.001	5	12.1%	0.172
Temporary employment	153	10.0%		106	9.3%		47	12.5%	
Self-employed	122	10.2%		53	10.6%		69	10.0%	
Unpaid family worker	37	8.5%		22	1.9%		15	6.6%	
Economically inactive population	370	11.8%		266	11.4%		104	12.9%	
Health status									
Excellent	151	4.7%	<0.001	98	4.0%	< 0.001	53	6.4%	<0.001
Fair	364	10.5%		236	9.4%		128	13.7%	
Poor	221	20.3%		162	20.4%		59	20.1%	
Chronic diseases									
No	106	2.0%	<0.001	61	1.6%	<0.001	45	2.8%	0.001
Yes	630	7.1%		435	6.6%		195	8.4%	
Disability									
No	661	8.6%	<0.001	438	7.7%	<0.001	223	10.9%	0.058
Yes	75	18.3%		58	18.3%		17	18.2%	

Note: We present weighted percentages of who answered ‘yes’ were asked for unmet dental needs owing to economic burdens by each category. Source: Korea Health Panel.

**Table 3:** Relationship between income level and unmet dental needs by the type of NHI beneficiary, 2011–2015.

Variables	Total						Employee Beneficiaries						Self-Employed Beneficiaries					
	Model 1			Model 2			Model 1			Model 2			Model 1			Model 2		
	OR	95% CI		OR	95% CI		OR	95% CI		OR	95% CI		OR	95% CI		OR	95% CI	
Health insurance type																		
Employee beneficiaries	1.00			1.00														
Self-employed beneficiaries	1.17	1.10–1.25		1.16	1.08–1.24													
Income level																		
5th quintile (highest)	1.00			1.00			1.00			1.00			1.00			1.00		
4th quintile	1.16	1.06–1.29		1.14	1.03–1.26		1.12	1.01–1.25		1.09	0.97–1.23		1.36	1.12–1.65		1.33	1.10–1.63	
3rd quintile	1.40	1.27–1.54		1.32	1.19–1.46		1.31	1.16–1.46		1.22	1.09–1.38		1.72	1.43–2.07		1.63	1.35–1.96	
2nd quintile	1.77	1.61–1.94		1.59	1.43–1.77		1.69	1.51–1.90		1.48	1.30–1.67		2.06	1.71–2.47		1.92	1.59–2.32	
1st quintile (lowest)	2.36	2.14–2.61		1.91	1.70–2.15		2.28	2.02–2.56		1.76	1.53–2.03		2.73	2.25–3.31		2.33	1.89–2.87	
Observation	35,415						25,037						10,378					

Model 1 was unadjusted; Model 2 was adjusted for gender, age, level of education, employment status, health status, chronic disease, and disability. Note: OR = odds ratio, CI = confidence interval. Odds ratio adjusted for unmeasured time-variant individual-level characteristics. Statistically significant *p*-values (<0.05) are presented in boldface. Source: Korea Health Panel.

**Table 4:** Relationship between income level and unmet dental needs due to the economic burden by the type of NHI beneficiary, 2011–2015.

Variables	Total						Employee Beneficiaries						Self-Employed Beneficiaries					
	Model 1			Model 2			Model 1			Model 2			Model 1			Model 2		
	OR	95% CI		OR	95% CI		OR	95% CI		OR	95% CI		OR	95% CI		OR	95% CI	
Health insurance type																		
Employee beneficiaries	1.00			1.00														
Self-employed beneficiaries	1.28	1.18–1.39		1.31	1.21–1.43													
Income level																		
5th quintile (highest)	1.00			1.00			1.00			1.00			1.00			1.00		
4th quintile	1.90	1.64–2.22		1.81	1.55–2.11		1.85	1.55–2.20		1.74	1.45–2.08		2.13	1.57–2.87		2.05	1.52–2.77	
3rd quintile	2.94	2.54–3.40		2.55	2.19–2.96		2.72	2.29–3.23		2.32	1.94–2.78		3.52	2.66–4.67		3.18	2.39–4.24	
2nd quintile	4.52	3.93–5.21		3.52	3.02–4.10		4.40	3.72–5.20		3.22	2.67–3.87		5.00	3.79–6.58		4.31	3.25–5.73	
1st quintile (lowest)	7.18	6.23–8.27		4.53	3.85–5.33		7.16	6.07–8.44		4.15	3.41–5.05		7.60	5.74–10.07		5.47	4.05–7.39	
Observation	32,919						23,258						9661					

Model 1 was unadjusted; Model 2 was adjusted for gender, age, level of education, employment status, health status, chronic disease, and disability. Note: OR = odds ratio, CI = confidence interval. Odds ratio adjusted for unmeasured time-variant individual-level characteristics. Statistically significant p-values (<0.05) are presented in boldface. Source: Korea Health Panel.

## Appendix A

**Table A1:** General characteristics of Korean adults aged 18+ according to health insurance type, 2011–2015.

Variables	2011			2012			2013			2014			2015		
	Total	Employee Beneficiaries	Self-Employed Beneficiaries	Total	Employee Beneficiaries	Self-Employed Beneficiaries	Total	Employee Beneficiaries	Self-Employed Beneficiaries	Total	Employee Beneficiaries	Self-Employed Beneficiaries	Total	Employee Beneficiaries	Self-Employed Beneficiaries
	N (Weighted %)	N (Weighted %)	N (Weighted %)	N (Weighted %)	N (Weighted %)	N (Weighted %)	N (Weighted %)	N (Weighted %)	N (Weighted %)	N (Weighted %)	N (Weighted %)	N (Weighted %)	N (Weighted %)	N (Weighted %)	N (Weighted %)
Total	7083 (100.0%)	4929 (70.2%)	2154 (29.8%)	7083 (100.0%)	4982 (70.8%)	2101 (29.2%)	7083 (100.0%)	5025 (71.4%)	2058 (28.6%)	7083 (100.0%)	5019 (71.8%)	2064 (28.2%)	7083 (100.0%)	5082 (71.6%)	2001 (28.4%)
Gender															
Male	2887 (41.8%)	2048 (41.4%)	839 (42.9%)	2887 (41.8%)	2048 (41.4%)	839 (42.9%)	2887 (41.8%)	2048 (41.4%)	839 (42.9%)	2887 (41.8%)	2048 (41.4%)	839 (42.9%)	2887 (41.8%)	2048 (41.4%)	839 (42.9%)
Female	4196 (58.2%)	3034 (58.6%)	1162 (57.1%)	4196 (58.2%)	3034 (58.6%)	1162 (57.1%)	4196 (58.2%)	3034 (58.6%)	1162 (57.1%)	4196 (58.2%)	3034 (58.6%)	1162 (57.1%)	4196 (58.2%)	3034 (58.6%)	1162 (57.1%)
Age															
18–44	2622 (43.6%)	1869 (45.0%)	753 (40.5%)	2448 (41.7%)	1782 (43.5%)	666 (37.3%)	2270 (40.3%)	1671 (42.2%)	599 (35.5%)	2100 (38.6%)	1569 (41.0%)	531 (32.4%)	1929 (37.4%)	1459 (39.8%)	470 (31.0%)
45–64	2801 (40.1%)	1885 (38.6%)	916 (43.7%)	2833 (41.6%)	1900 (39.4%)	933 (47.1%)	2885 (43.2%)	1943 (40.9%)	942 (49.0%)	2912 (43.9%)	1955 (41.3%)	957 (50.6%)	2960 (45.1%)	2038 (42.9%)	922 (51.0%)
More than 65	1660 (16.3%)	1175 (16.4%)	485 (15.8%)	1802 (16.7%)	1300 (17.1%)	502 (15.6%)	1928 (16.5%)	1411 (16.9%)	517 (15.5%)	2071 (17.5%)	1495 (17.7%)	576 (17.0%)	2194 (17.5%)	1585 (17.3%)	609 (18.0%)
Level of education															
≥College	2219 (36.5%)	1689 (39.9%)	530 (28.4%)	2232 (37.3%)	1715 (40.9%)	517 (28.5%)	2238 (38.4%)	1722 (41.8%)	516 (29.8%)	2241 (39.0%)	1714 (42.2%)	527 (30.9%)	2243 (40.2%)	1748 (43.8%)	495 (30.4%)
High school	2374 (35.2%)	1567 (33.2%)	807 (39.8%)	2361 (35.3%)	1565 (33.1%)	796 (40.7%)	2356 (35.4%)	1574 (33.3%)	782 (40.6%)	2353 (35.4%)	1589 (33.8%)	764 (39.3%)	2355 (35.4%)	1591 (33.4%)	764 (41.1%)
≤Middle school	2490 (28.3%)	1673 (26.8%)	817 (31.8%)	2490 (27.4%)	1702 (26.0%)	788 (30.8%)	2489 (26.2%)	1729 (24.9%)	760 (29.6%)	2489 (25.6%)	1716 (24.0%)	773 (29.8%)	2485 (24.4%)	1743 (22.8%)	742 (28.5%)
Income level															
5th quintile (highest)	1590 (25.4%)	1240 (28.7%)	350 (17.5%)	1625 (26.2%)	1274 (29.4%)	351 (18.5%)	1601 (26.7%)	1270 (29.9%)	331 (18.7%)	1613 (27.0%)	1265 (30.1%)	348 (19.4%)	1649 (27.9%)	1279 (30.6%)	370 (20.7%)
4th quintile	1615 (24.3%)	1183 (25.6%)	432 (21.3%)	1611 (24.5%)	1177 (25.6%)	434 (21.6%)	1625 (24.7%)	1212 (26.0%)	413 (21.3%)	1655 (25.7%)	1216 (26.8%)	439 (23.0%)	1623 (25.6%)	1206 (26.7%)	417 (22.6%)
3rd quintile	1532 (22.0%)	1019 (20.7%)	513 (25.2%)	1527 (21.9%)	1056 (21.3%)	471 (23.3%)	1544 (22.2%)	1026 (20.7%)	518 (25.9%)	1500 (21.6%)	1017 (20.6%)	483 (24.1%)	1497 (22.5%)	1033 (21.6%)	464 (24.6%)
2nd quintile	1387 (18.0%)	855 (15.6%)	532 (23.7%)	1389 (17.7%)	858 (14.8%)	531 (24.8%)	1363 (16.9%)	884 (15.0%)	479 (21.8%)	1351 (16.6%)	863 (14.4%)	488 (22.2%)	1346 (15.9%)	875 (13.4%)	471 (22.8%)
1st quintile (lowest)	959 (10.3%)	632 (9.4%)	327 (12.3%)	931 (9.7%)	617 (8.8%)	314 (11.7%)	950 (9.5%)	633 (8.4%)	317 (12.3%)	964 (9.0%)	658 (8.1%)	306 (11.3%)	958 (8.1%)	689 (7.7%)	279 (9.2%)
Employment status															
Permanent employment	1407 (23.3%)	1341 (31.6%)	66 (3.7%)	1373 (23.1%)	1324 (31.5%)	49 (2.8%)	1389 (23.7%)	1338 (32.1%)	51 (2.9%)	1374 (24.3%)	1346 (33.2%)	28 (1.5%)	1377 (25.2%)	1335 (33.5%)	42 (2.5%)
Temporary employment	1280 (18.9%)	882 (18.4%)	398 (20.0%)	1378 (20.8%)	983 (20.8%)	395 (21.0%)	1358 (20.6%)	991 (21.2%)	367 (19.3%)	1376 (20.7%)	1009 (21.1%)	367 (19.5%)	1397 (21.7%)	1056 (22.7%)	341 (19.1%)
Self-employed	1308 (16.9%)	598 (10.3%)	710 (32.5%)	1301 (16.8%)	575 (9.5%)	726 (34.4%)	1330 (17.2%)	606 (10.0%)	724 (35.4%)	1324 (17.1%)	565 (9.2%)	759 (37.2%)	1192 (15.7%)	504 (8.2%)	688 (36.2%)
Unpaid family worker	409 (5.0%)	219 (3.6%)	190 (8.2%)	421 (5.0%)	221 (3.5%)	200 (8.5%)	417 (4.8%)	218 (3.2%)	199 (8.8%)	413 (4.7%)	217 (3.1%)	196 (8.8%)	376 (4.4%)	195 (3.8%)	181 (9.1%)
Economically inactive population	2679 (35.9%)	1889 (36.0%)	790 (35.5%)	2610 (34.3%)	1879 (34.7%)	731 (33.4%)	2589 (33.6%)	1872 (33.6%)	717 (33.6%)	2596 (33.2%)	1882 (33.3%)	714 (33.0%)	2741 (33.0%)	1992 (33.0%)	749 (33.0%)
Health status															
Excellent	3177 (46.3%)	2212 (46.1%)	965 (46.6%)	3047 (45.4%)	2172 (46.2%)	785 (43.7%)	2840 (42.5%)	2.017 (42.9%)	823 (41.6%)	2823 (42.0%)	2030 (42.7%)	793 (40.3%)	2800 (44.3%)	2018 (44.6%)	782 (43.3%)
Fair	2960 (42.0%)	2064 (42.4%)	896 (41.1%)	2978 (42.1%)	2075 (41.6%)	903 (43.1%)	3254 (45.9%)	2317 (46.0%)	937 (45.5%)	3072 (44.3%)	2168 (44.3%)	904 (44.1%)	3212 (44.3%)	2291 (44.1%)	921 (44.8%)
Poor	946 (11.7%)	653 (11.5%)	293 (12.3%)	1058 (12.5%)	735 (12.2%)	323 (13.2%)	989 (11.6%)	691 (11.1%)	298 (12.9%)	1188 (13.7%)	821 (13.0%)	367 (15.6%)	1071 (11.4%)	773 (11.3%)	298 (11.9%)
Chronic diseases															
No	2473 (40.3%)	1707 (40.3%)	766 (40.1%)	2387 (39.5%)	1684 (40.0%)	703 (38.4%)	2163 (37.3%)	1542 (38.0%)	621 (35.5%)	2156 (37.6%)	1532 (38.3%)	624 (35.9%)	2138 (38.3%)	1537 (39.1%)	601 (36.0%)
Yes	4610 (59.7%)	3222 (59.7%)	1388 (59.9%)	4696 (60.5%)	3298 (60.0%)	1398 (61.6%)	4920 (62.7%)	3483 (62.0%)	1437 (64.5%)	4927 (62.4%)	3487 (61.7%)	1440 (64.1%)	4945 (61.7%)	3545 (60.9%)	1400 (64.0%)
Disability															
No	6695 (95.5%)	4659 (95.6%)	2036 (95.2%)	6686 (95.5%)	4692 (95.6%)	1994 (95.4%)	6678 (95.6%)	4727 (95.6%)	1951 (95.4%)	6671 (95.6%)	4706 (95.5%)	1965 (95.7%)	6654 (95.6%)	4748 (95.5%)	1906 (96.0%)
Yes	388 (4.5%)	270 (4.4%)	118 (4.8%)	397 (4.5%)	290 (4.4%)	107 (4.6%)	405 (4.4%)	298 (4.4%)	107 (4.6%)	412 (4.4%)	313 (4.5%)	99 (4.3%)	429 (4.4%)	334 (4.5%)	95 (4.0%)
Unmet dental care needs															
No	5827 (82.3%)	4119 (83.4%)	1708 (79.6%)	5772 (82.3%)	4113 (83.3%)	1659 (80.0%)	5617 (80.3%)	4025 (81.0%)	1592 (78.8%)	5859 (83.6%)	4199 (84.8%)	1660 (80.4%)	5878 (84.5%)	4258 (85.3%)	1620 (82.3%)
Yes	1256 (17.7%)	810 (16.6%)	446 (20.4%)	1311 (17.7%)	869 (16.7%)	442 (20.0%)	1466 (19.7%)	1000 (19.0%)	466 (21.2%)	1224 (16.4%)	820 (15.2%)	404 (19.6%)	1205 (15.5%)	824 (14.7%)	381 (17.7%)
Unmet dental care needs due to economic burden															
No	5827 (89.0%)	4119 (90.4%)	1708 (85.8%)	5772 (88.5%)	4113 (89.6%)	1659 (85.7%)	5617 (87.7%)	4025 (88.9%)	1592 (84.9%)	5859 (90.3%)	4199 (91.7%)	1660 (86.6%)	5878 (91.0%)	4258 (91.8%)	1620 (88.8%)
Yes	758 (11.0%)	463 (9.6%)	295 (14.2%)	848 (11.5%)	541 (10.4%)	307 (14.3%)	893 (12.3%)	582 (11.1%)	311 (15.1%)	731 (9.7%)	462 (8.3%)	269 (13.4%)	736 (9.0%)	496 (8.2%)	240 (11.2%)
